# CRISPR/Cas9: A Novel Weapon in the Arsenal to Combat Plant Diseases

**DOI:** 10.3389/fpls.2018.02008

**Published:** 2019-01-15

**Authors:** Ayan Das, Namisha Sharma, Manoj Prasad

**Affiliations:** National Institute of Plant Genome Research, New Delhi, India

**Keywords:** CRISPR/Cas9, guide RNA, protospacer adjacent motif, genome editing, pathogen- resistance, host susceptibility factor

## Abstract

Plant pathogens like virus, bacteria, and fungi incur a huge loss of global productivity. Targeting the dominant R gene resulted in the evolution of resistance in pathogens, which shifted plant pathologists’ attention toward host susceptibility factors (or S genes). Herein, the application of sequence-specific nucleases (SSNs) for targeted genome editing are gaining more importance, which utilize the use of meganucleases (MN), zinc finger nucleases (ZFNs), transcription activator-like effector-based nucleases (TALEN) with the latest one namely clustered regularly interspaced short palindromic repeats (CRISPR)/CRISPR-associated protein 9 (Cas9). The first generation of genome editing technologies, due to their cumbersome nature, is becoming obsolete. Owing to its simple and inexpensive nature the use of CRISPR/Cas9 system has revolutionized targeted genome editing technology. CRISPR/Cas9 system has been exploited for developing resistance against virus, bacteria, and fungi. For resistance to DNA viruses (mainly single-stranded DNA viruses), different parts of the viral genome have been targeted transiently and by the development of transgenic plants. For RNA viruses, mainly the host susceptibility factors and very recently the viral RNA genome itself have been targeted. Fungal and bacterial resistance has been achieved mainly by targeting the host susceptibility genes through the development of transgenics. In spite of these successes CRISPR/Cas9 system suffers from off-targeting. This and other problems associated with this system are being tackled by the continuous discovery/evolution of new variants. Finally, the regulatory standpoint regarding CRISPR/Cas9 will determine the fate of using this versatile tool in developing pathogen resistance in crop plants.

## Introduction

Plants are continuously being exposed to various pathogens including bacteria, fungi and viruses resulting in 20–40% yield loss globally (Savary et al., 2012; Borrelli et al., 2018). Dominant R-gene-mediated breeding has been the classical approach (Dangl et al., 2013) to achieve resistance against pathogens, which, due to strong selection pressure, resulted in the evolution of resistance among pathogens (Vleeshouwers et al., 2011; Win et al., 2012). Host susceptibility factors (or S genes) came out as alternatives, which are mainly negative regulators of immunity or host proteins, which, upon manipulation by a pathogen, support their growth (Langner et al., 2018). Over the past few years, new breeding techniques (NBTs) have been developed as alternatives to classical plant breeding for crop improvement including pathogen- resistance (Lusser and Davies, 2013; Nelson et al., 2018). NBT include the usage of sequence-specific nucleases (SSNs) such as meganucleases (MNs), zinc finger nucleases (ZFNs), transcription activator-like effector nucleases (TALENs), and clustered regularly interspaced short palindrome repeats (CRISPR)/CRISPR-associated protein 9 (Cas9), which have revolutionized targeted modifications of genomes. The requirement of sophisticated protein engineering rendered MN, ZFN, and TALEN techniques less practicable.

**FIGURE 1:**
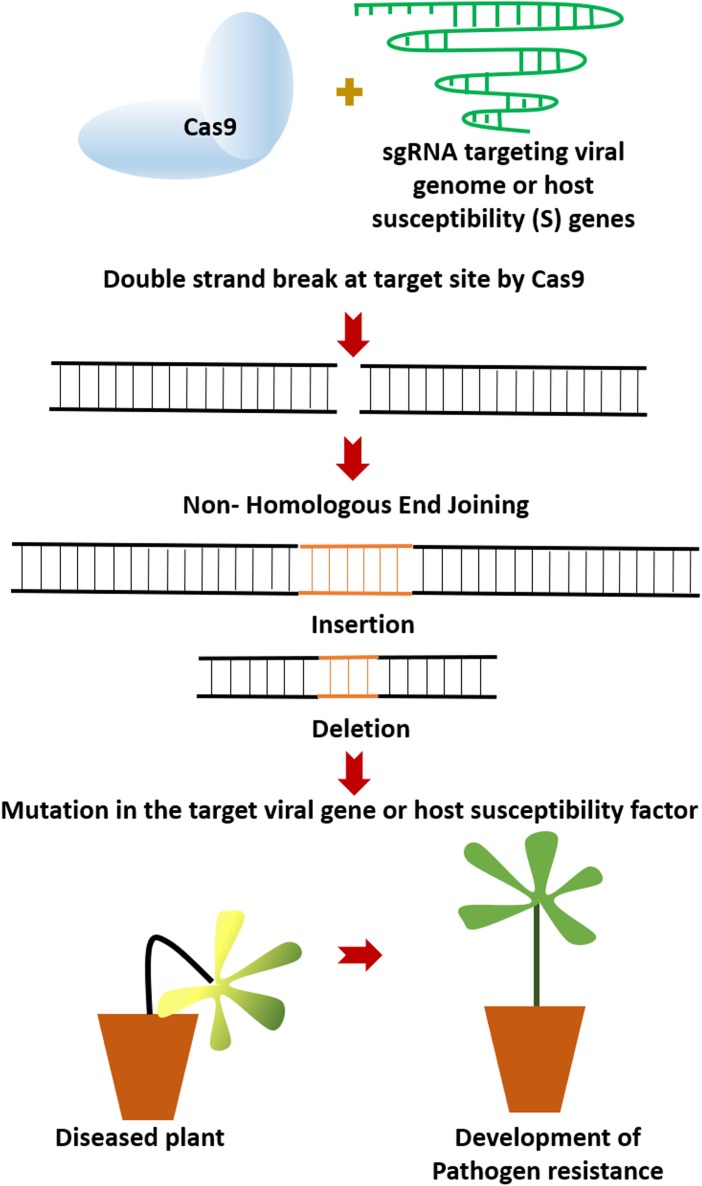
The CRISPR/Cas9 targeting and subsequent non- homologous end joining (NHEJ) process exploited for development of pathogen resistance in plants. SgRNA, single guide RNA.

The CRISPR-Cas9 system initially reported from *Streptococcus pyogenes* as class II bacterial adaptive immune system (Langner et al., 2018) is a two-component system consisting of the Cas9 nuclease and a customizable single guide RNA (sgRNA) (Khatodia et al., 2016). Additionally, it requires a protospacer adjacent motif (PAM) sequence (5′-NGG-3′) to induce double-stranded break (DSB) at the target site. DSBs can be repaired either by Homology-Directed Repair (HDR) or more frequently by non- homologous end joining (NHEJ) (Khatodia et al., 2016). Due to its error-prone nature, NHEJ leads to small indels (insertions/deletions) within the target region (Figure 1). This strategy of the CRISPR/Cas9 system has been exploited in plant pathology to target the S genes or the viral genomes. In this context, the mini-review will discuss the recent advances in crop protection against viral, fungal and bacterial pathogens using the CRISPR/Cas9 technology, the advantages, limitations, and possible ways for further improvement of this technology for better utilization in targeted genome editing.

## Achievements in Plant Virus Resistance Through CRISPR/Cas9 Technology

Utilization of the CRISPR/Cas9 system for the viral resistance has been executed by targeting either the viral genome or the host susceptibility factors (Table 1). Most of the CRISPR/Cas9 mediated viral resistance have been achieved by targeting the ssDNA of the geminiviruses (Ali et al., 2015, 2016; Baltes et al., 2015; Ji et al., 2015) with mono- or bi-partite genome containing the genes encoding proteins required for viral replication, movement, and suppressor of host defense machinery (Sharma and Prasad, 2017). Majority of the reports of targeting viral genes using CRISPR/Cas9 are either of transient type or through the development of transgenics in model plants like *Nicotiana benthamiana* and *Arabidopsis thaliana*.

**Table 1:** Major applications of CRISPR/Cas9 technology for viral, fungal, and bacterial resistance in plants.

Resistance against (name of the organism)	Target gene/intergenic region	Function of the gene/intergenic region	Plant species	Reference
**Viral resistance**				
BSCTV	CP, Rep, and IR	Rolling circle replication	*Nicotiana benthamiana* and *Arabidopsis thaliana*	Ji et al., 2015
BeYDV	Rep binding site, hairpin, invariant nonanucleotide sequence within the replication stem loop and Rep motifs I, II, and III	Rolling circle replication	*Nicotiana benthamiana*	Baltes et al., 2015
TYLCV, BCTV, MeMV	CP, RCR II motif of Rep and IR	Rolling circle replication	*Nicotiana benthamiana*	Ali et al., 2015
CLCuKoV, MeMV, TYLCV	CP, Rep, and IR	Rolling circle replication	*Nicotiana benthamiana*	Ali et al., 2016
TuMV	GFP, HC-Pro, CP	Viral replication	*Nicotiana benthamiana*	Aman et al., 2018
CMV, TMV	ORF1, 2, 3, CP and 3′ UTR	Viral replication	*Nicotiana benthamiana* and *Arabidopsis thaliana*	Zhang et al., 2018
CVYV, ZYMV, PRSV-W	eIF4E	Host susceptibility factor for viral translation	*Cucumis sativus*	Chandrasekaran et al., 2016
TuMV	eIF(iso)4E	Host susceptibility factor for viral translation	*Arabidopsis thaliana*	Pyott et al., 2016
RTSV	eIF4G	Host susceptibility factor for viral translation	*Oryza sativa* var. *indica* cv. IR64	Macovei et al., 2018
**Fungal resistance**				
Powdery mildew (*Blumeria graminis* f. sp. *tritici*)	*TaMLO-A1*	Host susceptibility (S) gene involved in powdery mildew disease	*Triticum aestivum*	Wang et al., 2014
Powdery mildew (*Oidium neolycopersici*)	*SlMlo1*	Host susceptibility (S) gene involved in powdery mildew disease	*Solanum lycopersicum*	Nekrasov et al., 2017
Powdery mildew (*Oidium neolycopersici*)	Exon-2, *Sl*PMR4	Host susceptibility (S) gene involved in powdery mildew disease	*Solanum lycopersicum*	Koseoglou, 2017
Rice blast disease (*Magnaporthe oryzae*)	*OsERF922*	Transcription factor involved in multiple stress responses	*Oryza sativa L. japonica* (var. Kuiku131)	Wang et al., 2016
**Bacterial resistance**				
Bacterial blight (*Xanthomonas oryzae* pv. *oryzae*)	SWEET13	Sucrose transporter	*Oryza sativa*	Zhou et al., 2015
*Pseudomonas syringae, Xanthomonas gardneri, X. perforans, Phytophthora capsici*	Exon-3, *Sl*DMR6–1,	Susceptibility factor in *Pseudomonas syringae pv. tomato* or *Phytophthora capsici* infection	*Solanum lycopersicum*	de Toledo Thomazella et al., 2016
*Pseudomonas syringae* pv. *tomato* DC3000	*Sl*JAZ2	Co-receptor for virulence factor coronatine (COR)	*Solanum lycopersicum*	Ortigosa et al., 2018
Fire blight (*Erwinia amylovora*)	DIPM-1, 2, 4	Host susceptibility factor for fire blight disease	*Malus domestica*	Malnoy et al., 2016


*BSCTV, beet severe curly top virus; BeYDV, bean yellow dwarf virus; TYLCV, tomato yellow leaf curl virus; BCTV, beet curly top virus; MeMV, Merremia mosaic virus; TRV, tobacco rattle virus; CLCuKoV, cotton leaf curl Kokhran virus; TuMV, turnip mosaic virus; CMV, cucumber mosaic virus; TMV, tobacco mosaic virus; CVYV, cucumber vein yellowing virus; ZYMV, zucchini yellow mosaic virus; PRSV-W, papaya ring spot mosaic virus-W; RTSV, rice tungro spherical virus; CP, coat protein; Rep, replication initiator protein; IR, intergenic region; GFP, green fluorescent protein; HC-Pro, helper component proteinase silencing suppressor; ORF, open reading frame; UTR, untranslated region; eIF4E, eukaryotic translation initiation factor 4E; eIF4G, eukaryotic translation initiation factor 4G; MLO, mildew resistant locus O; ERF922, ethylene responsive factor; SWEET, sugar will eventually be exported transporter; DMR6, Downy mildew resistance 6; PMR4, Powdery Mildew Resistance 4; JAZ2, Jasmonate ZIM-domain 2; DIPM-1, 2, 4, DspE-interacting proteins of Malus 1, 2, 4.*

The first report of exploitation of CRISPR/Cas9 system for geminivirus resistance came from Baltes et al. (2015) and Ji et al. (2015). Ji et al. (2015) first utilized the CRISPR/Cas9 system to develop beet severe curly top virus (BSCTV) resistance in *Arabidopsis* and *N. benthamiana* plants overexpressing sgRNA-Cas9. Baltes et al. (2015) demonstrated that transgenic *N. benthamiana* plants constitutively expressing Cas9 and sgRNA-Cas9 exhibit enhanced resistance against bean yellow dwarf virus (BeYDV) resulting in reduced viral load and symptoms.

Ali et al. (2015) delivered guide RNAs in Cas9 expressing *N. benthamiana* via tobacco rattle virus (TRV) vector targeting the viral capsid protein (CP), the RCRII motif of the replication protein (Rep) and the intergenic region (IR) of tomato yellow leaf curl virus (TYLCV). Guide RNA targeting the stem-loop sequence within the origin of replication in the IR was found to be the most effective. As the stem-loop sequence of the origin of replication in the IR is conserved in all geminiviruses, this system also provided resistance to other geminiviruses like a monopartite beet curly top virus (BCTV) and the bipartite Merremia mosaic virus (MeMV). Ali et al. (2016) further extended this work to show that targeting the non-coding IR results in durable resistance as it restricted the generation of virus variants capable of replication and movement, which was not achievable by targeting the coding sequences of geminiviruses. This observation is of great importance for future researchers while targeting the viral genome for long-term, durable resistance against viruses. This work also demonstrated successful utilization of the CRISPR/Cas9 system to develop resistance against Cotton leaf curl Kokhran virus (CLCuKoV).

As Cas9 from *S. pyogenes* can only edit double-stranded DNA, its initial application was limited to target the DNA viruses alone. Search for RNA editing nucleases led to the discovery of FnCas9 from *Francisella novicida* (Hirano et al., 2016; Green and Hu, 2017) and LwaCas13a (previously known as C2c2) from *Leptotrichia wadei* (Abudayyeh et al., 2017; Green and Hu, 2017). The first report of direct targeting of the genome of RNA viruses came from Zhang et al. (2018). The *N. benthamiana* and *Arabidopsis* plants expressing FnCas9 and sgRNA specific for cucumber mosaic virus (CMV) or tobacco mosaic virus (TMV) showed a significant reduction in virus accumulation and weakened symptom development. More importantly, the resistance was heritable, and the resultant progenies exhibited significantly reduced virus accumulation.

The other RNA endonuclease Cas13a has dual functions: processing of pre- CRISPR RNAs (crRNAs) and sequence-directed endonucleolysis of the target single-stranded RNA (Khan et al., 2018). Aman et al. (2018) employed CRISPR/Cas13a system in *N. benthamiana* to interfere with the RNA genome of turnip mosaic virus (TuMV). Targeting the HC-Pro and GFP sequences provided improved resistance than targeting the coat protein (CP) sequence. Successful multiplex targeting of the viral genome by utilization of the inherent ability of Cas13a to process the poly crRNA into individual crRNAs has also been exhibited (Aman et al., 2018). Although the RNA knockdown efficiency of another Cas13 family, namely Cas13b is greater than that of the Cas13a (Cox et al., 2017; Ying, 2018), there is no report yet regarding its utilization in a plant system.

Before the discovery of RNA-guided RNA editing systems, the only way to combat the RNA viruses was to target the host susceptibility factors for viral infection such as the eukaryotic translation initiation factor 4E (eIF4E), eIF(iso)4E, and eIF4G (Sanfacon, 2015). The eIF4E from plants was reported as a crucial host susceptibility component for viral infection and forms the largest group of recessive virus resistance genes in monocots and dicots (Ruffel et al., 2006; Hofinger et al., 2011). The eIF4E and eIF(iso)4E from tomato and melon exhibited recessive resistance against viruses (Mazier et al., 2011; Rodriguez-Hernandez et al., 2012). Targeting eIF4E of cucumber within non-homologous regions of exons 1 and 3 resulted in enhanced resistance against potyviruses such as cucumber vein yellowing virus, zucchini yellow mosaic virus and papaya ringspot mosaic virus-W in homozygous T_3_ lines (Chandrasekaran et al., 2016).

In a similar genome editing approach using CRISPR/Cas9 technology, Pyott et al. (2016) targeted the eIF(iso)4E locus in *Arabidopsis thaliana*. The resulting genome edited plants were resistant toward TuMV. Segregation of CRISPR/Cas9 transgene was observed in the T_2_ generation, and the resulting T_3_ homozygous lines exhibited morphologically normal phenotype. Macovei et al. (2018) developed tungro disease resistance [causal agent: rice tungro spherical virus (RTSV)] in susceptible rice cultivar IR64 by targeting translation initiation factor 4 gamma (eIF4G) gene.

The advantage of targeting host susceptibility genes is that it results in recessive resistance, which is more durable than dominant *R* gene-mediated resistance. The probable reason for this is that viruses endure a lower selection pressure impeding their evolution (Borrelli et al., 2018). A problem in targeting the susceptibility genes is that they are also required for translation of the host cells. Hence, although editing of eIF4E results in potyvirus resistance in lettuce, it also impairs with the physiology of the plant (Abdul-Razzak et al., 2009). In another study, disruption of *OsSEC3A* by CRISPR/Cas9 in rice resulted in enhanced resistance to *Magnaporthe oryzae* but it also impaired the normal growth of the plant resulting in a dwarf phenotype (Ma et al., 2018).

## Development of Fungal Resistance Via CRISPR/Cas9 Technology

Fungal resistance via CRISPR/Cas9 were mainly achieved till date by targeting the S genes like mildew resistance locus O (*MLO*), rice Ethylene Response Factor 922 (Table 1). The mildew resistance locus O (*MLO*) is the most widely known S gene locus. Since its identification in 1942 (Jørgensen, 1992), several mutants have been generated to provide resistance against powdery mildew in barley. *MLO* encodes a seven transmembrane domain-containing calmodulin binding protein located at the plasma membrane (Kim et al., 2002). Its role in susceptibility toward powdery mildew disease in monocot and dicot plants has also been confirmed (Kusch and Panstruga, 2017). Wang et al. (2014) targeted all three homoeoalleles- *MLO-A1*, *MLO-B1* and *MLO-D1* by TALEN and *TaMLO-A1* allele of exon 2 of bread wheat using CRISPR/Cas9 technology. Both the approaches were successful in generation of heritable resistance against powdery mildew caused by *Blumeria graminis* f. sp. *tritici*.

Powdery mildew in tomato is caused by Ascomycete fungus *Oidium neolycopersici* (Jones et al., 2001). Tomelo, a non-transgenic tomato variety resistant to *O. neolycopersici* has been developed by Nekrasov et al. (2017) using CRISPR/Cas9 technology. Targeting the *SlMlo1* locus by two sgRNAs resulted in the deletion of 48 bp in the said locus. Segregation of T-DNA was achieved by selfing of the T_0_ transformants, which was further confirmed by whole-genome Illumina sequencing. Besides *MLO*, other S genes associated with powdery mildew have also been identified from *Arabidopsis*. One such example is Powdery Mildew Resistance 4 (PMR4) which encodes for a callose synthase (Huibers et al., 2013). Koseoglou (2017) targeted its ortholog in tomato *SlPMR4* using CRISPR/Cas9 technology- deletion and rare inversion mutation were observed in the targeted exon-2. The resulting T_2_ progenies exhibited partial resistance against *O. neolycopersici.*

Rice *OsERF922* encodes an APETELA2/ethylene response factor (AP2/ERF) type transcription factor, which is strongly induced by *M. oryzae* (Liu et al., 2012). The identification of specific ERFs as negative regulators of plant immunity made them potential targets for genome editing (Langner et al., 2018). Targeting the OsERF922 gene using CRISPR/Cas9 technology in rice showed resistance to blast disease. The T_2_ mutant lines were similar to the wild-type rice plants with regard to several agronomic traits (Wang et al., 2016).

## Development of Resistance Against Bacteria Using CRISPR/Cas9 Technology

Compared to viral and fungal resistance few reports are available for utilization of CRISPR/Cas9 to combat bacterial diseases of crops (Table 1). The γ-proteobacterium, *Xanthomonas oryzae* pv. *oryzae* utilizes type III transcription-activator-like effectors (TALEs) to induce host gene expression resulting in host susceptibility. OsSWEET13, a sucrose transporter gene was identified as a susceptibility gene for *X. oryzae* pv. *oryzae* effector protein, PthXo2. Transfer of the *OsSWEET13* allele from *indica* rice IR24 to *japonica* rice Kitaake conferred disease susceptibility, whereas, mutations in the allele via CRISPR/Cas9 conferred resistance to bacterial blight (Zhou et al., 2015).

DMR6 (Downy mildew resistance 6) functions as a negative regulator of plant defense (Zeilmaker et al., 2015; Langner et al., 2018). de Toledo Thomazella et al. (2016) demonstrated that DMR6 ortholog *Sl*DMR6–1 is upregulated in tomato during infection with *Pseudomonas syringae* pv. *tomato* or *Phytophthora capsici* (Langner et al., 2018). Targeting exon-3 of *Sl*DMR6–1 resulted in mutated plants with a truncated version of *Sl*DMR6 showing broad-spectrum resistance against *Xanthomonas gardneri*, *X. perforans P. syringae* and *P. capsici* (de Toledo Thomazella et al., 2016; Langner et al., 2018).

*Pseudomonas syringae* pv. *tomato* (*Pto*) DC3000 is the causal agent of tomato bacterial speck disease. It produces coronatine (COR) which induces stomatal opening ensuing invasion of bacteria. In *Arabidopsis*, this stomatal response to COR is dependent on COR co-receptor AtJAZ2 (Jasmonate ZIM-domain-2). The truncated form of JAZ2 lacking the C-terminal Jas domain (JAZ2Δjas) prevent stomatal opening by COR (Gimenez-Ibanez et al., 2017). Ortigosa et al. (2018) identified ortholog of AtJAZ2 in tomato (*Sl*JAZ2), and it was targeted by CRISPR/Cas9 to generate dominant JAZ2 repressor- *Sl*JAZ2Δjas which prevented COR induced stomatal opening and provided resistance to biotrophic microbe *Pto* DC3000. Their experiment is also an example of successful uncoupling between the jasmonate (JA) and salicylate (SA) mediated defense pathways toward necrotroph and biotroph respectively. Effectual defense against biotrophs generally leads to increased susceptibility to necrotrophs and vice versa (Gimenez-Ibanez et al., 2017). As in this experiment, JA-signaling outside the stomata remained unaffected, *Sl*jaz2Δjas plants were also resistant to the necrotrophic fungi *Botrytis cinerea* which causes tomato gray mold.

The enterobacterium *Erwinia amylovora* causes fire blight disease in apple and other commercially important Rosaceae plants (Malnoy et al., 2016). The pathogenicity effector (DspE) of *E. amylovora* interacts with four leucine-rich-repeat, receptor-like serine/theonine kinases produced by DspE-interacting proteins of Malus (DIPM) genes- *DIPM 1, 2, 3, 4* (Borejsza-Wysocka et al., 2006). Malnoy et al. (2016) utilized the CRISPR/Cas9 system to target *DIPM 1, 2* and *4* genes in apple protoplast to develop resistance against fire blight disease. The experiment by Malnoy et al. (2016) also demonstrates successful direct delivery of CRISPR/Cas9 ribonucleoproteins (RNPs) (pre-assembled sgRNA/Cas9 complex) into plant protoplasts which has several benefits like rapid targeting efficiency, improved on-target and reduced off-target activity (Malnoy et al., 2016; Borrelli et al., 2018).

## Advantages and Limitations of CRISPR/Cas9 Technology

The main advantage of CRISPR/Cas9 technology is its inexpensiveness and ease of use. Unlike ZFN and TALEN, which is dependent on protein engineering, synthesis and validation (Joung and Sander, 2013; Voytas, 2013; Puchta and Fauser, 2014), here only the guide RNA needs to be designed. This single guide RNA (sgRNA) provides target-site specificity in CRISPR/Cas9 system (Jinek et al., 2012). Another advantage of CRISPR/Cas9 system compared to the first-generation genome editing techniques is the ability of multiplex genome editing, i.e., targeting multiple genes using a single construct (Murugan et al., 2017; Borrelli et al., 2018). Additionally, using the CRISPR/Cas9 system transgene-free genome edited plants can be obtained in very few generations (Khatodia et al., 2016; Langner et al., 2018). The efficiency of CRISPR/Cas9 system relies on the method of transformation. In plants generally routinely used methods are *Agrobacterium*-mediated transformation, biolistic transformation, and protoplast transformation. To perform CRISPR based homology-directed repair, the biolistic method is preferred over the other two methods (Baysal et al., 2016; Shi et al., 2017). Several factors such as the type of promoter used for driving the expression of Cas9 (e.g., 35S, rice ubiquitin promoter) and promoter driving sgRNA (e.g., rice snoRNA U3 promoter, *Arabidopsis* U6 promoter) also determines the targeted genome editing efficiency (Ma et al., 2016).

Like every other technique CRISPR/Cas9 system also has its limitations. Although less frequent in plants, CRISPR/Cas9 system suffers from off-target mutations (Langner et al., 2018). This can be tackled by using paired nickases where the RuvC domain of Cas9 is inactivated. As a result, it creates a nick instead of a double-strand break at the target site (Khatodia et al., 2016; Langner et al., 2018). Two nicks induced in close proximity ultimately produces a double-strand break (Hsu et al., 2014). In addition, the paired nickase system is also useful in high-efficiency HDR (Khatodia et al., 2016). The problem of off-targeting can also be tackled by the use of recently discovered CRISPR/Cpf1 from *Prevotella* and *Francisella* 1 (Cpf1) (Zetsche et al., 2015; Puchta, 2017; Zaidi et al., 2017) which creates a staggered double-strand break at the target site. Recent reports of genome editing by Cpf1 exhibited little to no off-target effects in rice (Tang et al., 2017; Xu et al., 2017). Additionally, Woo et al. (2015) reported that when ribonucleoproteins complexes or RNPs, instead of DNA were transfected into lettuce protoplasts, no off-target mutations were detected in the genome.

Another factor that limits the utilization of Cas9 is the PAM specificity. The stringent requirement of NGG motif immediately after the protospacer element limits targeting of the high AT-rich genome (Zetsche et al., 2015). The CRISPR/Cpf1 system mentioned earlier is also useful in this regard. Cpf1 recognizes a T-rich PAM sequence 5′-TTTN-3′ (or 5′-TTTV-3′; V = A, C, or G, in some cases) instead of 5′-NGG-3′. Cpf1 generates a staggered double-strand break with cohesive ends, which can also be useful for increasing the HDR efficiency (Zaidi et al., 2017). To tackle the problem of PAM specificity mutations have also been generated in the PAM-interacting domain of wild-type *Sp*Cas9 (Kleinstiver et al., 2015) which recognize alternative PAM sequences like NGCG, NGAG (Anders et al., 2016; Langner et al., 2018).

## Future Perspectives

Targeted genome editing by CRISPR/Cas9 can yield desired disease resistant traits within a very short period which cannot be achievable by traditional breeding methods (Borrelli et al., 2018; Langner et al., 2018). When resistance achieved via dominant R genes is amenable to be overcome by the adaptive potential of the pathogens, targeting the host susceptibility factors seems to be a smarter alternative. The availability of the genome sequences of economically important crops and their transcriptomics datasets can be useful in the identification of new S genes (Zaidi et al., 2018). However, the CRISPR/Cas9 technology is still in its juvenile phase- field trial of the genome edited crops is limited (Shi et al., 2017) which will be essential to check the durability of the incurred pathogen resistance. The regulatory issues regarding genome edited crops will play an important role in this regard. The United States Department of Agriculture (USDA) does not regulate genome edited plants which could otherwise have been developed through traditional breeding techniques (Jaganathan et al., 2018). On the other hand, in the European Union, genome edited crops are currently subject to regulations as genetically modified (GM) organisms (Callaway, 2018). Although safety issues regarding the application of CRISPR/Cas9 technology must be examined by scientific means and considering the associated practical and societal aspects (Bechtold, 2018), regulatory standpoint in favor of this promising technology will assist in its proper dissemination leading to better crop management.

## Author Contributions

All authors listed have made a substantial, direct and intellectual contribution to the work, and approved it for publication.

## Conflict of Interest Statement

The authors declare that the research was conducted in the absence of any commercial or financial relationships that could be construed as a potential conflict of interest.
